# Interface-to-Surface Transition Induced Topological Hall Effect in 2-Dimensional SrRuO_3_ Integrated on Silicon

**DOI:** 10.34133/research.1079

**Published:** 2026-01-21

**Authors:** Qinglong Wang, Bin He, Jinrui Guo, Jianping Zhang, Yue Han, Huan Liu, Weidong Wang, Shengshi Li, Weiming Lü, Shishen Yan

**Affiliations:** ^1^ Spintronics Institute, School of Physics and Technology, University of Jinan, Jinan 250022, China.; ^2^ School of Physics, Harbin Institute of Technology, Harbin 150001, China.

## Abstract

The topological Hall effect (THE), a transport signature emerging from chiral spin textures induced by structural symmetry breaking and the Dzyaloshinskii–Moriya interaction (DMI), represents a rich frontier in condensed matter physics with promising applications in spintronic devices. To enhance the DMI and thereby induce THE in SrRuO_3_ (SRO), we introduce a structural-symmetry-breaking strategy that disrupts the Ru–O termination at the rigid substrate interface. This disruption triggers a transition from a rigid epitaxial interface to a freestanding membrane with unsaturated surface bonds, resulting in asymmetric surface terminations (Sr–O on top and Ru–O at the bottom). Unlike its rigid counterpart, which shows no detectable THE, the freestanding SRO exhibits a pronounced THE signal, persisting up to 100 K while preserving high crystallinity and electronic coherence. The ability to generate robust THE in transferable oxide membranes has direct implications for next-generation spintronics, offering compelling prospects for creating low-power magnetic memory and logic devices based on chiral spin textures.

## Introduction

The topological Hall effect (THE), a hallmark of real-space Berry curvature effects [1–3], serves dual roles in modern condensed matter physics. Fundamentally, it provides critical insights into exotic topological phases, such as skyrmion lattices [4] and chiral spin liquids [5], that challenge conventional band theory. Technologically, THE enables transformative spintronic applications, including racetrack memories [6,7], spin–orbit torque oscillators [8,9], neuromorphic computing [10], etc., by permitting precise manipulation of chiral spin textures with exceptional energy efficiency and scalability. The emergence of THE requires a delicate balance between spatial inversion symmetry and time-reversal symmetry breakings for Dzyaloshinskii–Moriya interaction (DMI) and magnetic order [11–13], respectively. Theoretical frameworks posit an optimized DMI strength of *D*∼(*JK*)^1/2^, where *D*, *J*, and *K* correspond to the DMI, Heisenberg exchange, and magnetic anisotropy energies. Thus, unit-cell-level strategies are always necessary, such as existing strain engineering, stoichiometric control, and heterointerface design in THE materials (e.g., SrRuO_3_ [SRO] [14–16], Fe_3_Sn_2_ [17], and bilayer CrI_3_ [18]).

**Fig. 1.**
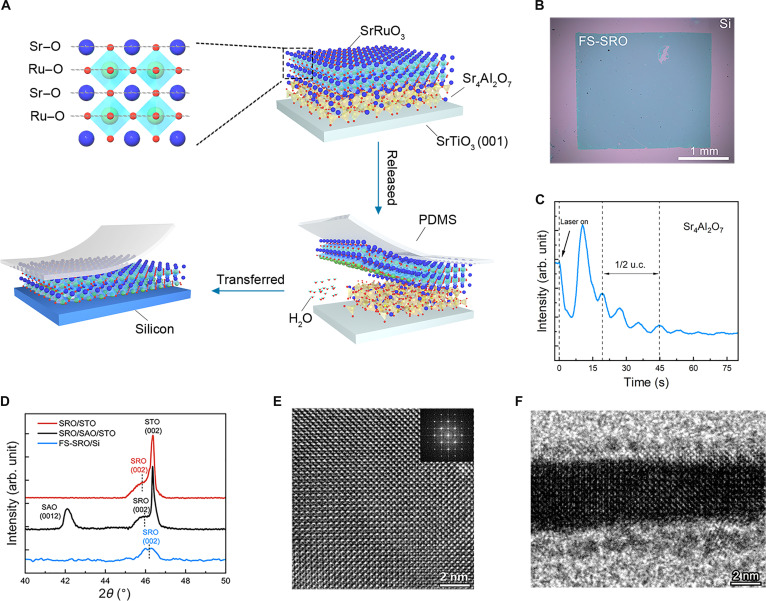
Preparation and structural characterization of FS-SRO membranes. (A) Schematic illustration of the fabrication of FS-SRO membranes. The sacrificial SAO layer is dissolved in deionized water to release the SRO films with the support of PDMS. FS-SRO membranes are transferred onto the Si substrate. (B) Optical microscopy image of FS-SRO (3.6 nm) on a Si substrate. (C) Time-dependent RHEED intensity oscillation profiles during the growth of SAO films. A period of three oscillation peaks indicates the deposition of 1/2 unit cell (u. c.). (D) XRD 2θ–ω scans of SRO (12 nm) films, SRO (12 nm)/SAO (12 nm) heterostructures on STO (001), and FS-SRO (12 nm) on Si substrate. (E) Plan-view HRTEM image of FS-SRO membrane. The inset shows the fast Fourier transformation of FS-SRO. (F) Cross-sectional HRTEM image of FS-SRO membrane on Si.

Specifically, SRO, a THE pronounced complex oxide, inherently facilitates structural symmetry breaking due to the intrinsic interfacial and electronic asymmetries between Sr–O and Ru–O atomic layers at the subunit-cell level (Fig. 1A). In SRO, the Sr–O bonding is predominantly ionic, while Ru–O exhibits mixed covalent–ionic character, accompanied by a shorter Ru–O bond length compared to Sr–O [19]. These inherent differences make the RuO_6_ octahedra highly susceptible to tilting and distortion, thereby promoting the emergence of THE. However, simultaneously exposing both Sr–O and Ru–O as surface terminations remains challenging, since high-quality low-dimensional SRO structures are typically epitaxial, requiring one of these planes to be clamped by the substrate. It is expected that overcoming this limitation enables the realization of SRO with spontaneous structural symmetry breaking while preserving high crystallinity and electronic coherence. Moreover, by circumventing interfacial defects, chemical interdiffusion, and magnetic dead layers in conventional heterostructures [20,21], the artifactual mimicry of topological Hall signatures can be avoided. It is noteworthy that SRO is highly sensitive to electronic phase homogeneity and that even minor inhomogeneities can introduce competing positive and negative contributions to the anomalous Hall effect (AHE), as previously reported [22–24]. Equally compelling is the transferability of these freestanding THE-active SRO layers, offering a critical capability for designing novel spin-electronic device paradigms.

In this study, we present the distinct THE in the freestanding SRO (FS-SRO) membrane that was released from epitaxial SRO/Sr_4_Al_2_O_7_ (SAO)/SrTiO_3_ (STO) by water dissolution of the sacrificial SAO interlayer. By disrupting the Ru–O termination at the SRO/SAO interface, the SRO transitions from a rigid heterostructure with mixed covalent–ionic bonds to a freestanding film with unsaturated dangling bonds. This process gives rise to exposed, asymmetric surface terminations, Sr–O on the top and Ru–O on the bottom, distinct from conventional approaches for tuning the THE. Notably, whereas epitaxially rigid SRO (RG-SRO) on STO exhibits no detectable THE, the FS-SRO transferred onto silicon demonstrates a pronounced THE that persists up to 100 K. We attribute this emergent THE to the asymmetric Ru–O- and Sr–O-terminated surfaces in FS-SRO, which enhance structural symmetry breaking, amplify the DMI, and thereby induce the observed topological Hall response. Our demonstration of a freestanding, flexible, and Si-compatible THE layer not only provides a versatile platform for fundamental research on strain-tunable topological spin textures but also establishes a direct pathway to integrate nonvolatile skyrmion-based logic and memory devices into mainstream silicon technology.

## Results

### High-quality FS-SRO membranes with crystalline integrity

As depicted in Fig. 1A, FS-SRO membranes of varying thicknesses were fabricated by dissolving a SAO sacrificial interlayer in SRO/SAO/STO heterostructures. During the etching process, the lower Sr–O termination layer was selectively removed (see Supplementary Note), after which the released FS-SRO membranes were transferred onto Si substrates using a high-precision transfer platform. This method ensured accurate alignment and preserved membrane integrity with minimal damage. The large-scale FS-SRO membrane (3.6 nm) exhibited no wrinkles or cracks, as illustrated in Fig. 1B, which is particularly advantageous for testing the structural information and ferromagnetic performance on Si substrates. The growth rate and layer-by-layer growth of the SAO sacrificial layer were monitored in situ via reflection high-energy electron diffraction (RHEED), as shown in Fig. 1C. The designed thickness of the SAO sacrificial layer in our work is about 12 nm, which can ensure the efficient large-area exfoliation of the ultrathin SRO membranes. Meanwhile, the SAO layer exhibits an exceptionally high dissolution rate [25,26], demonstrating the SRO layer to fully detach from the rigid substrate within 30 min. Figure 1D shows x-ray diffraction (XRD) patterns of the 2θ–ω scans of SRO/STO (001) films, SRO/SAO/STO (001) heterostructures, and FS-SRO on Si. On the basis of the position of the (0012) diffraction peak of the SAO layer and the (002) diffraction peaks of the SRO layers, we determined the out-of-plane lattice parameters *c* for SAO and SRO layers grown on the STO substrate. The calculated *c* of the SAO layer is approximately 25.743 Å, and the *c* of the SRO layer deposited on the STO and SAO/STO substrates is about 3.958 and 3.946 Å, respectively. Owing to the lattice mismatch between bulk SRO [19] and STO substrates, the in-plane compressive strain in SRO films will enlarge the *c* of SRO layers. The insertion of SAO layers will reduce compressive stress, thus decreasing the *c* of SRO layers. The dissolution of the SAO layer can result in the release of stress in the SRO layer imposed by the STO substrate. This is confirmed by XRD measurements, which reveal that the *c* of the FS-SRO membrane is about 3.928 Å. To thoroughly investigate the microstructural characteristics of the FS-SRO membrane, we conducted high-resolution transmission electron microscopy (HRTEM) analysis on both cross-sectional and plan-view samples (Fig. 1E and F). The HRTEM images of the FS-SRO (~4.8 nm) confirm their high crystallinity and the well-defined perovskite structure. Quantitative lattice analysis can confirm nearly identical in-plane (~3.922 Å) and out-of-plane (~3.932 Å) lattice parameters, closely matching the results obtained from XRD calculations (Fig. 1B). Combining the results from XRD and TEM, we demonstrated the successful preparation of high-quality single-crystal 2-dimensional FS-SRO membranes.

### Enhanced ferromagnetism in FS-SRO membranes

**Fig. 2.**
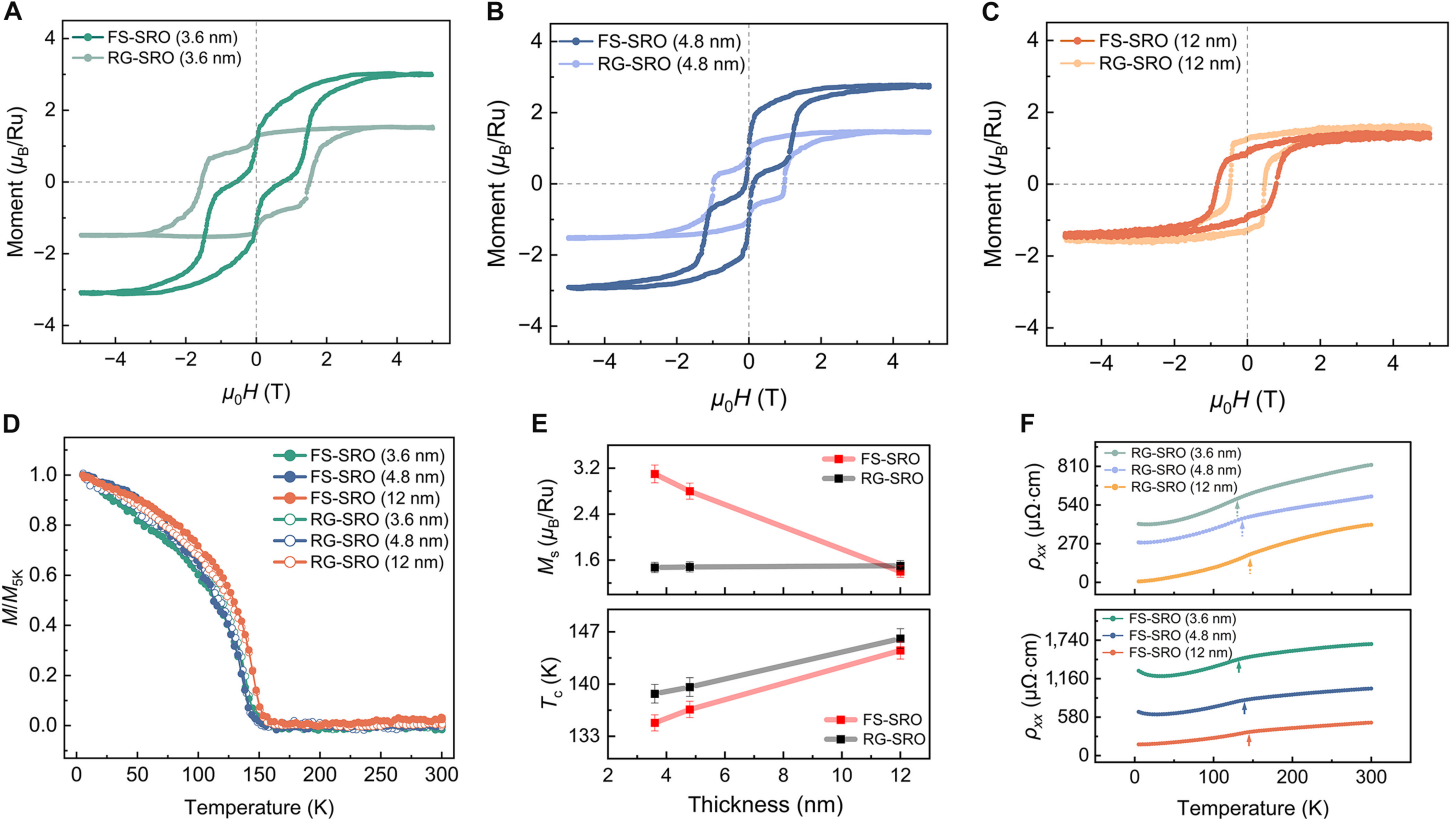
Magnetic and transport properties of RG-SRO and FS-SRO membranes. (A to C) The *M*–*H* of RG-SRO and FS-SRO are presented for 3 different thicknesses: 3.6, 4.8, and 12 nm at 5 K. Magnetic fields were applied along the out-of-plane direction. (D) Normalized *M*–*T* curves of RG-SRO and FS-SRO with various thicknesses: 3.6, 4.8, and 12 nm. The *M*–*T* curves were measured with an out-of-plane magnetic field of 1,000 Oe. (E) Variation of *M*_s_ and *T*_c_ with SRO thickness for RG-SRO films and FS-SRO membranes. (F) Comparison of temperature-dependent longitudinal resistivity *ρ_xx_* for RG-SRO and corresponding FS-SRO membranes.

To systematically investigate the evolution of magnetic and transport characteristics, we conducted comparative measurements across RG-SRO and FS-SRO membranes, including out-of-plane magnetic hysteresis (*M*–*H*) loops, field-cooled magnetization (*M*–*T*) curves, and temperature-dependent resistivity (*ρ_xx_*–*T*) curves. Figure 2A to C shows the out-of-plane *M*–*H* loops of RG-SRO and FS-SRO with different thicknesses at 5 K. The RG-SRO and FS-SRO samples exhibit well-saturated *M*–*H* loops with an applied magnetic field of ±5 T. It is noteworthy that *M*–*H* curves (<12 nm) exhibit double-loop characteristics with distinct coercivities (*H*_c_), demonstrating the magnetic phase existence (hard and soft magnetic phases). The emergence of double hysteresis loops in SRO originates from the oxygen vacancy during the growth process [27–30]. To further elucidate the origin of the double magnetic hysteresis loops in RG-SRO films, we systematically performed preannealing and postannealing treatments on substrates under controlled oxygen partial pressures, followed by comprehensive magnetic and transport characterizations (Fig. S5A to F). After oxygen annealing, the SRO films show only one ferromagnetic phase and a decreased saturation magnetization. This observation suggests that oxygen vacancies are responsible for the presence of dual magnetic phases. Meanwhile, the strong oxygen affinity of aluminum in the SAO/SRO heterostructure drives interfacial redox reactions, generating more oxygen vacancies in the FS-SRO membranes [31–33]. Therefore, the ultrathin (<12 nm) FS-SRO membranes exhibit larger magnetic moments than those of RG-SRO films. As illustrated in Fig. 2E, the saturation magnetization (*M*_s_) of FS-SRO membranes increases rapidly with the decrease in thickness. The *M*_s_ of the RG-SRO films is nearly independent of thickness and is approximately 1.5 *μ*_B_/Ru, consistent with reported values with low spin configuration of *t*_2*g*_ (3↑, 1↓) [19,34–36]. In particular, the *M*_s_ of the FS-SRO membrane with 3.6 nm is about 3.1 *μ*_B_/Ru, indicating that the high-spin configuration is induced in our 2-dimensional SRO membrane [37].

The spin-state transition is not only related to oxygen vacancies but may also be associated with the unique interfacial crystal structure of the freestanding membrane. Figure 2D presents the *M*–*T* curves of the RG-SRO films and FS-SRO membranes with varying thicknesses. For all SRO samples, a decrease in magnetization is observed with increasing temperature, accompanied by a sharp transition from ferromagnetic to paramagnetic behavior. The Curie temperature (*T*_c_) was determined via differentiation analysis of the *M*–*T* curves, specifically identified from the extremum point in the d*M*/d*T* versus *T* profile (Fig. S2). The *T*_c_ of the FS-SRO membranes and RG-SRO films exhibits a pronounced dependence on the modulation thickness. As the thickness of SRO films increases from 3.6 to 12 nm, the *T*_c_ of RG-SRO and FS-SRO exhibits a gradual increase from 138 to 146 K and 135 to 145 K, respectively. As displayed in Fig. 2F, the samples were further characterized by transport measurements of longitudinal resistivity. A characteristic thickness-dependent transition emerges, manifesting progressive suppression of both ferromagnetic ordering and metallic conduction characteristics with diminishing film thickness. Notably, FS-SRO membranes with thicknesses of 3.6 and 4.8 nm exhibit a metal–insulator transition temperature (<40 K), providing compelling evidence for progressive electron localization driving the system toward the insulating states [15]. Strong localization of 4d in FS-SRO membranes could increase the resistance and induce the metal–insulator transition. The distinct magnetic properties observed between FS-SRO and RG-SRO indicate fundamental differences in their electronic structure, originating from interfacial variations in spin configuration, chemical stoichiometry, and strain status.

### Emergent THE in FS-SRO membranes

**Fig. 3.**
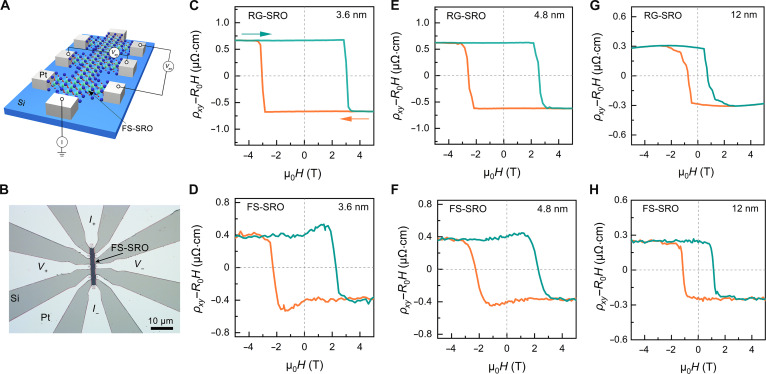
Hall transport properties of RG-SRO films and FS-SRO membranes. (A) Schematic of the Hall bar device for an FS-SRO film. *I*_+_ and *I*_−_ represent the applied current. *V_xx_* and *V_xy_* represent the measured longitudinal and transverse voltages, respectively. (B) Optical image of a Hall bar device for an FS-SRO membrane on the Si substrate. (C to H) Magnetic-field-dependent Hall resistivity *ρ_xy_* of RG-SRO and FS-SRO membranes with various thicknesses at 5 K. The *H* is applied along the out-of-plane direction. The ordinary Hall components (*R*_0_*H*) were subtracted.

To further systematically probe thickness-dependent transport evolution, we performed Hall resistivity measurements on RG-SRO and FS-SRO membranes to examine the electrical transport properties. Figure 3A and B shows the schematic and optical microscopy image of the typical Hall bar devices with platinum electrodes. The Hall resistivity of SRO can be expressed as$\;\rho_{\mathrm{xy}}=R_{0}H+R_{S}M+\rho_{\mathrm{xy}}^{T}$, where first term on the right side represents the ordinary Hall effect, which is proportional to out-of-plane magnetic field *H*. The negative ordinary Hall backgrounds provide evidence that the carriers are n type. The second term corresponds to the AHE, where *R*_S_ and *M* are the anomalous Hall coefficient and out-of-plane magnetization. The third term $\rho_{\mathrm{xy}}^{T}$ denotes the topological Hall resistivity that is typically associated with originates from magnetic skyrmions, chiral domain walls, or other noncollinear spin textures with chirality [38,39]. As shown in Fig. 3C to H, after subtracting the linear ordinary Hall term, all samples exhibit sign-reversed hysteresis loops in Hall resistivity *ρ_xy_*, confirming the existence of a negative *R*_s_. AHE hysteresis loops are observed in RG-SRO films at all thicknesses, consistent with previous literature [19]. In particular, as shown in Fig. 3D and F, distinct Hall anomalies emerge near ±*H*_c_ at 5 K for FS-SRO membranes with thicknesses of 3.6 and 4.8 nm. The observed hump signatures could be attributed to the term $\rho_{\mathrm{xy}}^{T}$. In addition, as the thickness of the FS-SRO membrane increases, the intensity of the Hall anomalies decreases monotonically, vanishing completely at 12 nm. To further validate the reproducibility of the Hall anomalies, we measured the Hall resistance as a function of magnetic field for 3 devices on the FS-SRO with 4.8 nm (Fig. S3). The consistent observation of emergent THE-like behavior in all devices implies that the freestanding state and its associated lattice structure can stabilize magnetic domain configurations in the ultrathin SRO membranes (Fig. S4A to C). More interestingly, these electrical/magnetic properties are exhibited in an isolated single-layer SRO without clamping or proximity effect from substrate or superlattice [16,40].

**Fig. 4.**
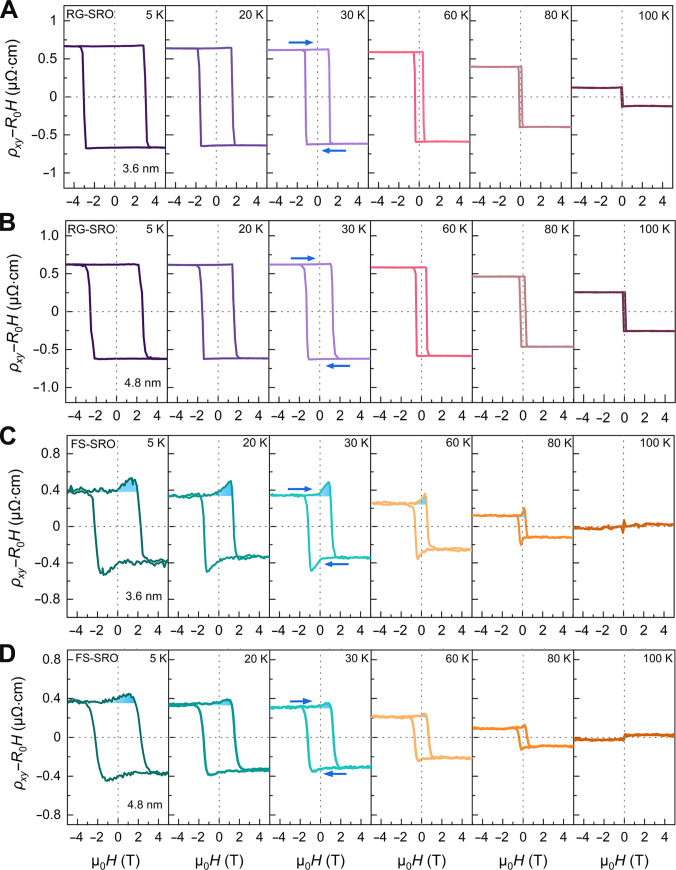
Hall resistivity of RG-SRO films and FS-SRO membranes. Magnetic field dependence of the Hall resistivity *ρ_xy_* of the RG-SRO films with 3.6 nm (A) and 4.8 nm (B) at various temperatures. Magnetic field dependence of the Hall resistivity *ρ_xy_* of the FS-SRO membranes with 3.6 nm (C) and 4.8 nm (D) at various temperatures. Blue arrows represent sweeping directions of the magnetic field. The ordinary Hall term was subtracted.

An alternative theoretical framework proposes a 2-channel AHE mechanism involving the superposition of opposite Hall polarities to explain the observed transport anomalies [22–24,41]. While our FS-SRO membranes exhibit *M*–*H* loops with 2 distinct magnetic orders, the pronounced hump in the *ρ_xy_*–*H* loops would seem to be attributed to a 2-channel AHE mechanism (Fig. 3D and F). However, rigid samples with very similar *M*–*H* loop profiles show no such anomalies in their *ρ_xy_*–*H* loops (Fig. 3C, E, and G). This discrepancy indicates that the 2-channel model cannot fully explain the topological Hall-like behavior. To investigate the origin of Hall anomalies, Fig. 4 presents the thickness- and temperature-dependent Hall resistivity hysteresis for RG-SRO and FS-SRO, with blue arrows indicating magnetic field sweep direction. For rigid samples, the Hall resistivity exhibited conventional AHE behavior without hump-like features. In contrast, freestanding samples demonstrated a distinct hump superimposed on the AHE hysteresis loop over a broad temperature range from 5 to 100 K (Fig. 4C). Notably, Hall anomalies consistently emerge below the AHE sign-reversal temperature in FS-SRO membranes (Fig. 4C and D). This contrasts with reported 2-channel AHE behavior in other SRO systems, where anomalies appear only in a narrow temperature window between the AHE sign-reversal temperature and *T*_c_ [42–44]. The Hall anomalies observed in FS-SRO recall the characteristic signatures of the intrinsic THE previously reported in ultrathin SRO films and SRO-based heterostructures [14–16].

### Manipulation of the THE by current and field orientation

**Fig. 5.**
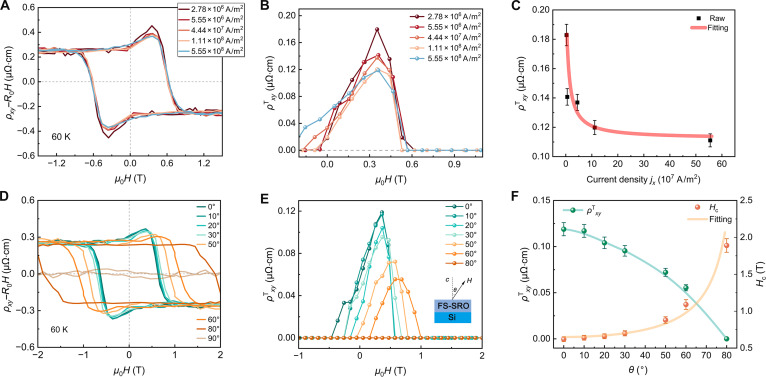
Current- and field-orientation dependence of THE in FS-SRO membranes. (A) Magnetic-field-dependent *ρ_xy_* of FS-SRO membranes (3.6 nm) at 60 K under various current density and (B) topological Hall resistivity$\;\rho_{\mathrm{xy}}^{T}\;$versus *μ*_0_*H*. (C)$\;\rho_{\mathrm{xy}}^{T}\;$as a function of increasing current density *j_x_*. The red solid line represents nonlinear fitting based on equation 1/*j_x_*. (D) $\rho_{\mathrm{xy}}\;$versus *μ*_0_*H* of the FS-SRO membranes (3.6 nm) at 60 K under various magnetic field canting angles *θ* and (E) topological Hall resistivity$\;\rho_{\mathrm{xy}}^{T}\;$versus *μ*_0_*H*. (F)$\;\rho_{\mathrm{xy}}^{T}\;$and *H*_c_ as a function of various field canting angles *θ*. The yellow solid curve represents nonlinear fitting based on equation 1/cos(*θ*).

On the other hand, as illustrated in Fig. 5A and B, the $\rho_{\mathrm{xy}}^{T}\;$ exhibit a progressive suppression with increasing longitudinal current density *j_x_* between 2.78 × 10^6^ and 5.55 × 10^8^ A/m^2^. Figure 5C shows inverse proportionality between the $\rho_{\mathrm{xy}}^{T}$ and *j_x_*, described by the scaling relation: $\;\rho_{\mathrm{xy}}^{T}\left(j\right)\propto 1/j_{x}$. To explain this current-dependent THE, we first provide a concise overview of skyrmion dynamics. Spin-polarized current applies a force on the magnetic spin structure via the spin–transfer torque mechanism, a phenomenon that has been widely studied in ferromagnetic systems [45,46]. Moving skyrmions can generate emergent electric fields [47]: $\;E=-P_{e}^{q^{e}}v_{s}\times b_{e}$, where *P*_e_ signifies the degree of spin polarization of the charge carriers, *q*^e^ represents the emergent charge that arises from the spin configurations of these charge carriers, including spin-up and spin-down, **v**_s_ is the drift velocity of skyrmions, and **b**_e_ is the emergent magnetic field generated by skyrmions. This transverse emergent electric field counteracts the additional Hall voltage, thereby suppressing the topological Hall resistivity, a hallmark manifestation of the skyrmion Hall effect (SkHE) [48,49]. Subsequently, the functional relationship between current density and topological Hall resistivity can be established through the constitutive relation [50]:$\;\rho_{\mathrm{xy}}^{T}\left(j\right)=E_{y}/j_{x},$ where *E_y_* denotes the net transverse emergent electric field arising from skyrmions [47]. At sufficiently high *j_x_*, the back action of the current becomes strong enough to overcome the pinning force of the skyrmion domains, causing the skyrmions to begin to move [4,47]. Meanwhile, high *j_x_* may destroy skyrmions by driving them toward nanotracks edges [51,52]. Our results are consistent with the proposed mechanism of current-driven skyrmion motion, suppressing the THE as discussed.

In materials with pronounced magnetic skyrmions, $\rho_{\mathrm{xy}}^{T}\;$should be strongly dependent on the canting angle of the magnetic field [53,54]. Figure 5D to F systematically demonstrates the angular dependence of THE under magnetic field inclinations ranging from out-of-plane (*θ* = 0°) to in-plane (*θ* = 90°). Both $\rho_{\mathrm{xy}}^{T}$ and the *H*_c_ exhibit strong angular dependence: $\rho_{\mathrm{xy}}^{T}$ decreases progressively with increasing canting angle *θ* and disappears at *θ* = 80°, while *H*_c_ follows characteristic 1/cos(*θ*) scaling (Fig. 5F). Furthermore, this suppression is consistent with skyrmion systems such as La_0.825_Sr_0.175_MnO_3_ [55] and CaMnO_3_/CaIrO_3_/CaMnO_3_ [56] trilayer structure, and the topological charge (*Q*) of skyrmions was transformed into zero when the magnetic field is tilted toward the in-plane orientation. Following the established relationship [56]:$\;\rho_{\mathrm{xy}}^{T}\propto Q$ , this phase transition consequently diminishes the THE. Our FS-SRO shows a wide phase diagram region of the THE signatures under the field tilting up to *θ* = 80°. In addition, as shown in Fig. S7, AHE loops of RG-SRO (3.6 nm) measured under varying current densities and magnetic field orientations exhibit robustness of maximum *ρ_xy_*, providing further evidence that the THE observed in FS-SRO originates from skyrmions, rather than the artifact of 2-channel AHE.

## Discussion

### Structural origin of THE in FS-SRO membranes

**Fig. 6.**
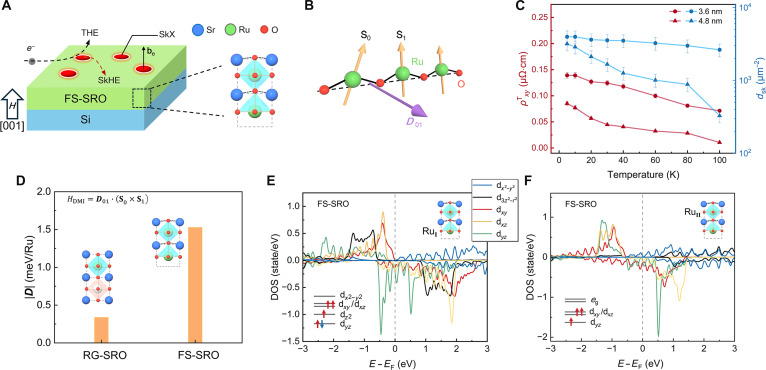
The generation of Ru–O-terminated surfaces and the emergence of DMI in FS-SRO membranes. (A) Schematic of the magnetic structure in the FS-SRO membrane. The etching of the Sr–O termination layer induces inversion symmetry breaking. (B) The DMI vector *D*_01_ is perpendicular to the plane defined by Ru–O–Ru. (C) Temperature dependence of $\rho_{\mathrm{xy}}^{T}\;$and *d*_sk_ for FS-SRO membranes. (D) DFT calculations of DMI strength (|*D*|) for the RG-SRO (SRO/STO) and FS-SRO. Calculated PDOS of the Ru_I_ (E) and Ru_II_ (F) atoms in the FS-SRO. The inset is a schematic diagram of the electron configuration of the d orbital of the Ru atom.

In summary, the observed THE in FS-SRO, including its characteristic hump, current-dependent suppression, and sensitivity to field orientation, provides strong evidence for stabilizing current-sensitive chiral spin textures such as skyrmions. To elucidate the connection between THE and magnetic spin texture, we investigate the microscopic origin of the DMI in the FS-SRO membranes. The stabilization of chiral spin textures in the strongly correlated oxide systems typically requires strong DMI from spin–orbit coupling (SOC) and inversion symmetry breaking [12,13]. The *ρ_xy_*–*H* loops of the RG-SRO layer with the varying concentration of oxygen defects (Fig. S6) indicate that, in our SRO, oxygen defects alone are insufficient to induce measurable THE. In FS-SRO membranes, a selective etching of Sr–O layers during sacrificial layer dissolution creates asymmetric Ru–O-terminated surfaces (Fig. 6A). This lattice distortion naturally breaks inversion symmetry, providing a possible way to generate the DMI. The Hamiltonian of the DMI is defined as follows: *H*_DMI_ = ***D***_01_ · (**S**_0_ × **S**_1_), where the DMI vector is given by ***D***_01_ = *D***r**_01_ × **z**. Here, **r**_01_ and **z** respectively represent pointing from **S**_0_ to **S**_1_ and unit vectors along the [001] axis. As shown in Fig. 6B, ***D***_01_ should be perpendicular to the Ru–O–Ru chains [11,13]. DMI stabilizes skyrmions in the FS-SRO, leading to the observations of the THE and SkHE. The evolution of skyrmion density (*d*_sk_) with sample thicknesses provides important information about the origin of skyrmions. Because of the strong interaction between local *M* in skyrmions and spin of charge carriers, the skyrmion density can be expressed as follows: $d_{\mathrm{sk}}=e\rho_{\mathrm{xy}}^{T}/R_{0}hP_{e}$, where *e* and *h* denote elementary charge and Planck constant, respectively. For SRO, we estimate *P*_e_ = −10 ± 5% according to previous reports [15,19]. The $\rho_{\mathrm{xy}}^{T}$ and *d*_sk_ reaches maximum at 5 K and decreases gradually with the increasing temperature (Fig. 6C), indicating that thermal fluctuations suppress skyrmion stability. Furthermore, the marked decay of *d*_sk_ with increasing FS-SRO thickness suggests that the skyrmions stabilization mechanism originates near the lower surface.

On this basis, we conducted density functional theory (DFT) 0o988calculations comparing lattice and electronic structures of RG-SRO and FS-SRO. The structural schematic diagrams and computational details are presented in Fig. S8 and Materials and Methods. It can be observed that the RG-SRO films greatly retain the structural features of bulk SRO, except for a buckling distortion in the surface Sr_II_–O_II_ layer, with the Sr_II_ atoms displaced downward, while the RuO_6_ octahedra remain intact. In contrast, FS-SRO undergoes more pronounced structural symmetry breaking, wherein the original RuO_6_ octahedra are transformed into RuO_5_ square pyramids due to the removal of the underlying Sr–O layer (Fig. S8). As listed in Table S1, compared to the RG-SRO, the difference between Ru_I_–O_I_ and Ru_II_–O_I_ bond lengths in FS-SRO is notably increased (*δ* = 0.29Å), and the diagonal Sr_I_–O_I_–Sr_I_ angle is concurrently decreased to 165.28°. As shown in Fig. 6D, DFT calculations revealed that the FS-SRO with the Ru–O-terminated surfaces shows larger degree of lattice distortion than the RG-SRO. Meanwhile, the DMI strength (|***D**|*) of FS-SRO is about 1.53 meV/Ru, approximately 5 times larger than RG-SRO (0.34 meV/Ru). The enhanced |***D**|* in FS-SRO membranes directly stabilize magnetic skyrmions, accounting for the observed THE. To further investigate the role of oxygen vacancies, as illustrated in Fig. S3A. The calculated DMI strength |***D***| for this defective structure, shown in Fig. S3B, increases to 2.00 meV/Ru. This enhancement can be attributed to the additional lattice distortion induced by oxygen vacancies at the bottom surface (Fig. S10). These results suggest that while oxygen vacancies alone are insufficient to trigger THE, appropriate concentration can further enhance the DMI strength in FS-SRO.

This structural symmetry breaking profoundly alters the magnetic properties of SRO. The magnetic moment calculated by DFT (Table S1) reveals that localized moments for Ru_I_ and Ru_II_ atoms in FS-SRO (RG-SRO) are found to be 2.58 *μ*_B_ (1.36 *μ*_B_) and 1.77 *μ*_B_ (1.43 *μ*_B_), respectively. In FS-SRO, the formation of RuO_5_ square pyramids crystal field at the Ru–O-terminated surface results in the splitting of the *e*_g_ orbitals into d_*x*2−*y*2_ and d_*z*2_. Projected density of states (PDOS) in Fig. 6E and F shows that the energy of d_*z*2_ orbital of Ru_I_ at Ru–O-terminated surfaces is lowered below the Fermi level, falling even below the d*_yz_* level. Moreover, in the absence of the Sr–O termination, the spin-down electron from the d*_yz_* orbital of Ru_II_, previously located near the Fermi level and involved in conduction, becomes transferred to the lower d_*z*2_ orbital of Ru_I_. Furthermore, because of the close energy proximity between the d_*z*2_ and d*_xy_*/d*_xz_* orbitals, the 3 electrons now preferentially align their spins parallel. This stabilizing high-spin configuration of Ru_I_ (d^5^, *S* = 3/2) and Ru_II_ (d^3^, *S* = 3/2), confirming the existence of high-spin states in FS-SRO compared to low-spin states of RG-SRO (d^4^, *S* = 1) in Fig. S11. This electronic reconstruction substantially enhances the *M*_s_ in the FS-SRO, which is consistent with our experimental results (Fig. 2). The Ru_I_ magnetic moment is slightly lower than expected 3.00 *μ*_B_. This discrepancy arises from the hybridization between the Ru_I_ d and O p orbitals, which induces a localized magnetic moment of ~0.30 *μ*_B_ on the oxygen atom. Furthermore, by analyzing the layer-resolved SOC energy difference (*E*_SOC_), as illustrated in Fig. S12A, b, we find that the DMI in both systems primarily originates from the Ru_I_ atom, since the *E*_SOC_ of O atoms is negligible. We have compared our system with SRO-based materials in Table S2. Most reported systems exhibit relatively low saturation magnetization, suggesting that conventional tuning approaches have limited influence on the spin state. In contrast, our method not only achieves a high saturation magnetization of up to 3.1 μB/Ru but also yields a substantial DMI strength. Meanwhile, the freestanding structure allows for seamless integration with silicon-based technologies, facilitating the development of next-generation spintronic devices.

In this work, we have successfully fabricated large-area 2-dimensional SRO membranes of varying thickness using a water-soluble SAO sacrificial layer from epitaxial SRO/SAO/STO heterostructures. We demonstrate that the ultrathin FS-SRO membranes integrated on silicon exhibit a pronounced THE over wide temperature range and under tilted magnetic field, which is attributed to the emergence of the noncollinear skyrmions. This phenomenon originates from the asymmetric Ru–O-terminated surface in the FS-SRO membrane, which substantially enhances structural symmetry breaking and the DMI strength, thereby inducing the THE. Furthermore, compared with rigid films, ultrathin FS-SRO membranes (<12 nm) stabilize high spin states with a large magnetic moment. These findings not only provide critical insights into the THE properties of 2-dimensional SRO materials but also establish a foundation for the development of next-generation spintronic devices based on silicon-integrated oxide membranes.

## Materials and Methods

### Preparation of epitaxial SRO films

The series of SRO films and SRO/SAO heterostructures was epitaxially grown on STO (001) single-crystal substrates by pulsed laser deposition (PLD) using a KrF excimer laser (*λ* = 248 nm). The STO substrates were subjected to an etching process utilizing buffered hydrofluoric acid and annealed in an ambient atmosphere at 1,000 °C for 90 min to achieve an atomically flat and TiO_2_-terminated surface. All SRO films were grown under an oxygen partial pressure of 1 × 10^−1^ torr at a temperature of 700 °C, and the laser repetition rate was set at 2 Hz. The water-soluble sacrificial SAO layers (~12 nm) were grown in an oxygen atmosphere of 5 × 10^−5^ torr at a temperature of 700 °C using a 2-Hz laser frequency, and growth was monitored via RHEED. After deposition, all samples were postannealed at growth temperature for 15 min under an oxygen pressure of 1 × 10^−1^ torr.

### Fabrication of FS-SRO membranes and structural characterizations

To fabricate highly crystalline FS-SRO membranes, the sacrificial SAO layer in the epitaxial heterostructures was selectively etched with deionized water. Upon the complete dissolution of the sacrificial layer, the film was exfoliated and adhered to polydimethylsiloxane (PDMS). FS-SRO membranes were transferred on Si substrate using the transfer stage with heating and a precise displacement controller (E1-T, Metatest Corporation, China). The crystal structures of the rigid, FS-SRO membranes and SRO/SAO heterostructures were investigated by XRD (Rigaku, SmartLab 3KW). The thickness, morphology, and crystal structures of FS-SRO membranes are characterized by TEM (F20, Tecnai) and atomic force microscopy (MFP-3D Origin+).

### Electrical transport and magnetic measurements

Longitudinal and transverse transport data at low temperature were measured using a Physical Properties Measurement System (PPMS; Quantum Design) on standard Hall bars. Photolithography and Ar ion milling were used to pattern the SRO films into the Hall bar geometry. The Pt films were deposited onto the Hall bar as contact electrodes via PLD. Transport characterizations, *M*–*H* loops, and *M*–*T* curves were measured using PPMS. The magnetic field was applied along the out-of-plane direction. Hall measurements were performed with the magnetic field sweeping up to ±5 T. *M*–*H* loops were obtained by sweeping the magnetic field up to ±5 T.

### DFT calculations

First-principles calculations were performed using the projected augmented wave method, as implemented in the Vienna ab initio simulation package (VASP) [57–59]. The generalized gradient approximation (GGA) in the form of Perdew–Burke–Ernzerhof (PBE) functional was adopted to describe the exchange–correlation interaction [60]. To treat the strong correlations of the Ru 4d orbitals, the GGA + *U* scheme was used, with the Hubbard parameters *U* and *J* set to 2.5 and 0.4 eV, respectively [61–63]. All structures were optimized until the force on each atom was less than 0.001 eV/Å, and the convergence criterion for energy was 10^−6^ eV. The plane-wave cutoff energy was set to 500 eV. To sample the Brillouin zone, Γ-centered *k* meshes of 19 × 19 × 1 for both RG-SRO and FS-SRO, respectively. A vacuum space of >20 Å was applied along the *z* direction to eliminate interactions between periodic images. The DMI strength was calculated on the basis of the chirality-dependent total energy difference approach in the presence of SOC using noncollinear calculations in VASP [64]. The 4 × 1 × 1 supercell was constructed for the RG-SRO and FS-SRO systems. The clockwise (CW) and anticlockwise (ACW) spin configurations of Ru atoms in various SRO films are illustrated in Fig. S9. The DMI energy is defined as *H*_DMI_ = ***D***_ij_ · (**S**_i_ × **S**_j_). The DMI strength was then calculated via the energy difference between CW and ACW spin configurations, expressed as *D* = (*H*_CW_ − *H*_ACW_)/*n*. In our systems, *n* equals 8.

## Data Availability

All data needed to evaluate the conclusions in the paper are present in the paper and/or the Supplementary Materials.

## Supplementary Materials

Supplementary 1 Supplementary Note Figs. S1 to S12 Tables S1 and S2
