# Supplementary material for: Interface-to-Surface Transition Induced Topological Hall Effect in 2-Dimensional SrRuO3 Integrated on Silicon
Source: Research (Wash D C). 2026 Jan 21;9:1079. doi: 10.34133/research.1079 (PMC12820467; doi:10.34133/research.1079)
Supplement: Supplementary 1 — Supplementary Note Figs. S1 to S12 Tables S1 and S2 [file research.1079.f1.docx]

**Supplementary Materials**

**Interface-to-surface Transition Induced** **Topological Hall Effect in Two-dimensional SrRuO_3_ Integrated on Silicon**

Qinglong Wang^1†^, Bin He^1†*^, Jinrui Guo^1^, Jianping Zhang^1^, Yue Han^2^, Huan Liu^2^, Weidong Wang^1^, Shengshi Li^1*^, Weiming Lü^1,2*^, and Shishen Yan^1*^

*^1^ Spintronics Institute, School of Physics and Technology, University of Jinan, Jinan 250022, China.*

*^2^ School of Physics, Harbin Institute of Technology, Harbin 150001, China.*

* Corresponding authors.

E-mail addresses: sdy_heb@ujn.edu.cn; [sdy_liss@ujn.edu.cn](mailto:sdy_liss@ujn.edu.cn); [weiminglv@hit.edu.cn](mailto:weiminglv@hit.edu.cn); shishenyan@sdu.edu.cn

† Qinglong Wang, and Bin He contributed equally to this work.

**Supplementary Note: Mechanism of Selective Etching and Surface Termination**

Here, we elucidate the formation mechanism of the RuO_2_ surface terminations. During the layer-by-layer growth of SRO on SAO, since the SrO surface terminations of SAO are already present, SRO grows in an alternating sequence of RuO_2_ and SrO layers, with the SrO layer being shared between SRO and SAO. The strong bonding strength at the ABO_3_/SAO interface implies that a higher ionization energy is required for the hydrolysis of SAO[1]. However, due to SAO’s water solubility, the Sr-O ionic bonds at the SAO/SRO interface are more prone to breaking. Furthermore, the high oxygen affinity of Al-based materials promotes the formation of oxygen vacancies at the SRO interface. The resulting Sr^2+^ ions from broken Sr-O bonds enhance the thermodynamic driving force for cation hydration, thereby increasing the interfacial solubility[2-4].

In contrast, the Ru-O bonds, stabilized by covalent interactions due to *d*-*p* orbital hybridization between Ru^4+^ and O^2-^, remain relatively unaffected by SAO. After selective etching of the SrO termination, the exposed RuO_2_ layer undergoes a slight atomic rearrangement to minimize its surface energy, thereby enhancing lattice distortion. Owing to the excellent chemical stability of SRO, the resulting RuO_2_ surface terminations can remain stable under ambient conditions.


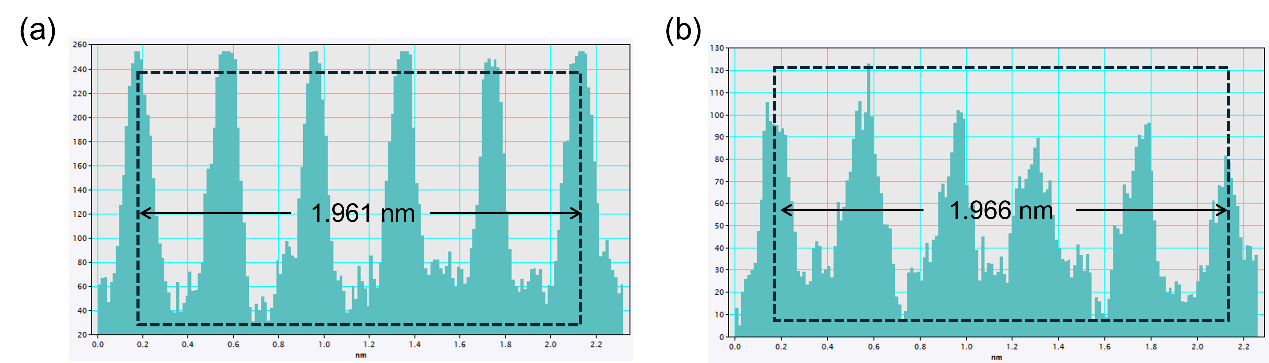


**

 Fig. S1.** (a) Line profiles of in-plane HRTEM image of FS-SRO along pseudo-cubic [100] or [010]. (b) out-of-plane line profiles of out-of-plane HRTEM image along pseudo-cubic [001].

**Fig. S2.** dM/dT curves obtained from the field-cooled (FC) curves measured with an out-of-plane (and cooling) field of 1000 Oe for RG-SRO and FS-SRO membranes.


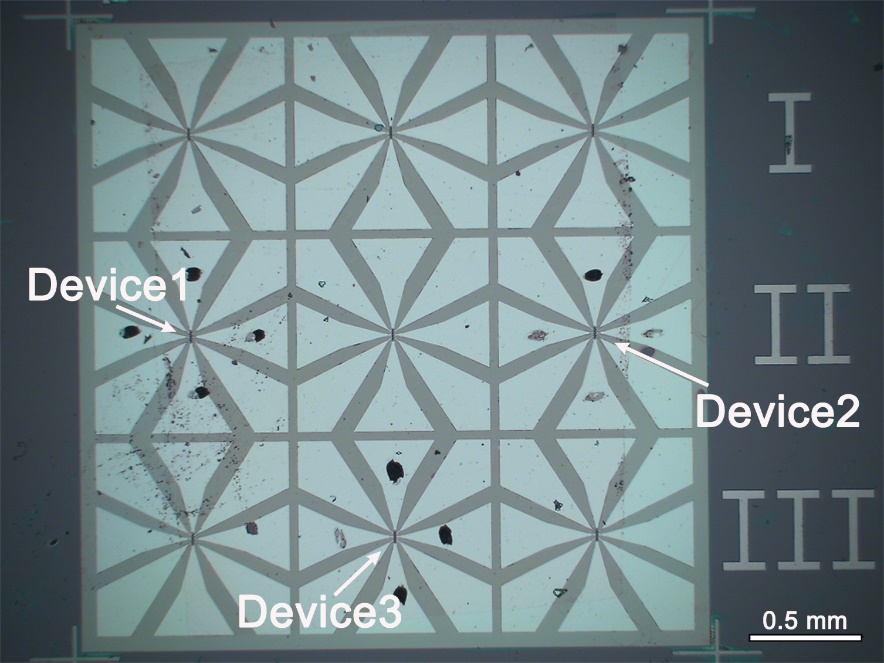


**Fig. S3.** Optical microscopy image of Hall devices array for FS-SRO (4.8 nm) membrane on Si substrate, with measurement channels labeled Device1, Device2, and Device 3.

**
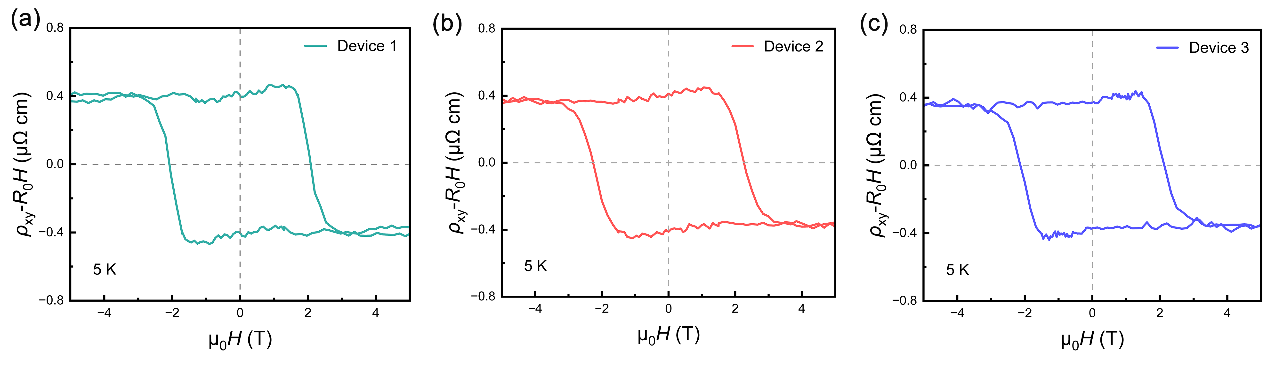
**

**Fig. S4.** Hall resistivity ρ_xy_ as a function of magnetic H for FS-SRO (4.8 nm), measured on Device 1, Device 2, and Device 3.


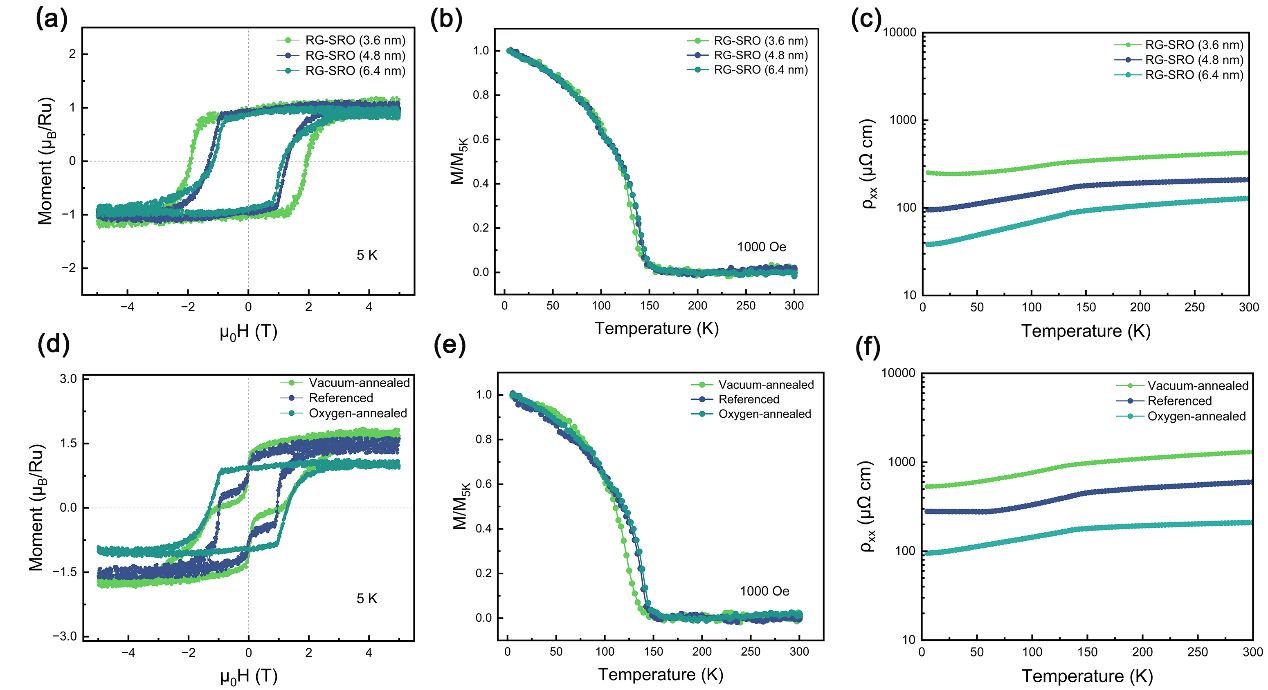


**Fig. S5. Oxygen annealing control of magnetic and transport properties in RG-SRO films. (a)** M-H loops of RG-SRO films with various thicknesses grown on pre-annealed STO substrates (0.1 Torr, 700 ℃), followed by post-annealing under oxygen pressures (4 Torr). **(b)** Normalized *M*-*T* curves of RG-SRO with an out-of-plane magnetic field (1000 Oe) and **(c)** longitudinal resistivity *ρ*_xx_-*T* curves. **(d)** M-H loops of RG-SRO film (4.8 nm) with different annealed conditions at 5 K. **(e)** Normalized *M*-*T* curves and **(f)** longitudinal resistivity *ρ*_xx_-*T* curves.

We systematically investigate oxygen vacancy engineering in RG-SRO films through controlled pre-annealing and post-annealing treatments under tailored oxygen partial pressures. This approach enables precise regulation of vacancy concentrations, yielding controllable regulation of magnetic phase transitions and AHE. We fabricated RG-SRO films with various thicknesses of 3.6, 4.8 and 6.4 nm. These films underwent pre-annealing at 700°C under 0.1 Torr oxygen partial pressure, followed by post-annealing at 4 Torr, respectively. This sequential annealing strategy successfully stabilized single magnetic phases, as evidenced by the M-H loops in **Fig. S5a**. Thickness-dependent trends show Tc ​increasing from 135 K to 142 K (**Fig. S5b**) and longitudinal resistivity ρ_xx_ decreasing (**Fig. S5c)** with increasing thickness.

Furthermore, we conducted a comparative study of RG-SRO films (4.8 nm) under distinct annealing protocols: (1) Vacuum-annealed film (pre-annealed at 10^-7^ Torr, post-annealed at 0.1 Torr): Enhanced *M*_s_ of 1.7 *μ*_B_/Ru (**Fig. S5d**) and elevated *ρ*_xx_ (**Fig. S5f**) confirm vacancy-induced spin polarization. (2) Referenced film (no pre-annealing, post-annealed at 0.1 Torr): Intermediate properties. (3) Oxygen-annealed film (pre-annealed at 0.1 Torr, post-annealed at 4 Torr): Near-stoichiometric behavior that exhibits single-loop magnetic hysteresis with few vacancies. Crucially, all samples display conventional AHE behavior analogous to bulk SRO (**Fig. S6a-c**), conclusively demonstrating that oxygen vacancies alone, while creating magnetic inhomogeneity, are insufficient to induce the topological Hall effect. This rules out the two-channel AHE mechanism as the origin of the hump-like feature.

*
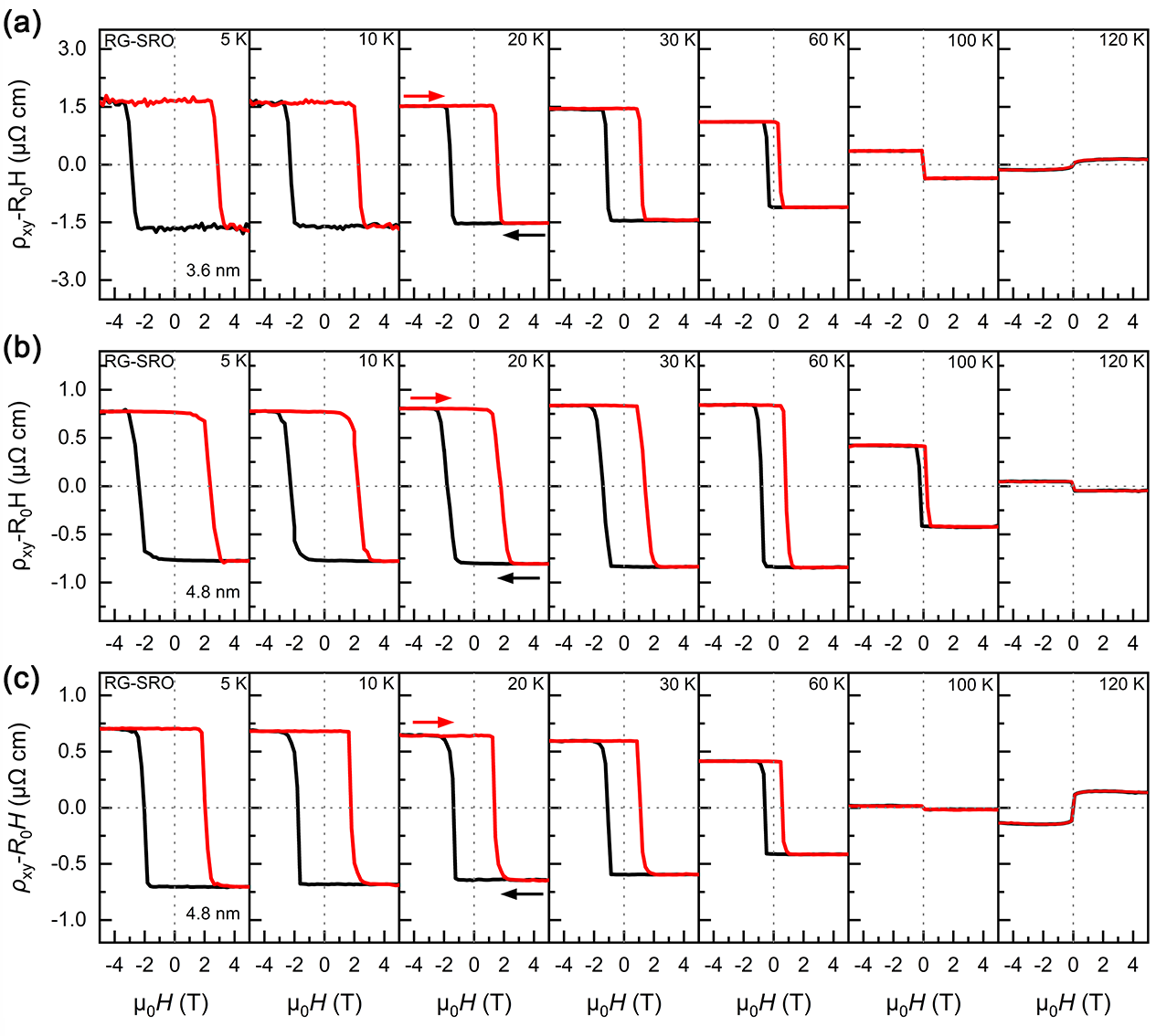
*

**Fig. S6. Oxygen annealing control of Hall resistivity in RG-SRO films. (a), (b)** Magnetic field dependence of the Hall resistivity *ρ*_xy_ of the RG-SRO films (3.6 and 4.8 nm) grown on pre-annealed STO substrates (0.1 Torr, 700 ℃), followed by post-annealing under oxygen pressures (4 Torr). **(c)** *ρ*_xy_-*H* loops of the RG-SRO films (4.8 nm) grown on pre-annealed STO substrates under high vacuum atmospheres (10^-7^ Torr, 700 ℃), followed by post-annealing under oxygen pressures (0.1 Torr).

**
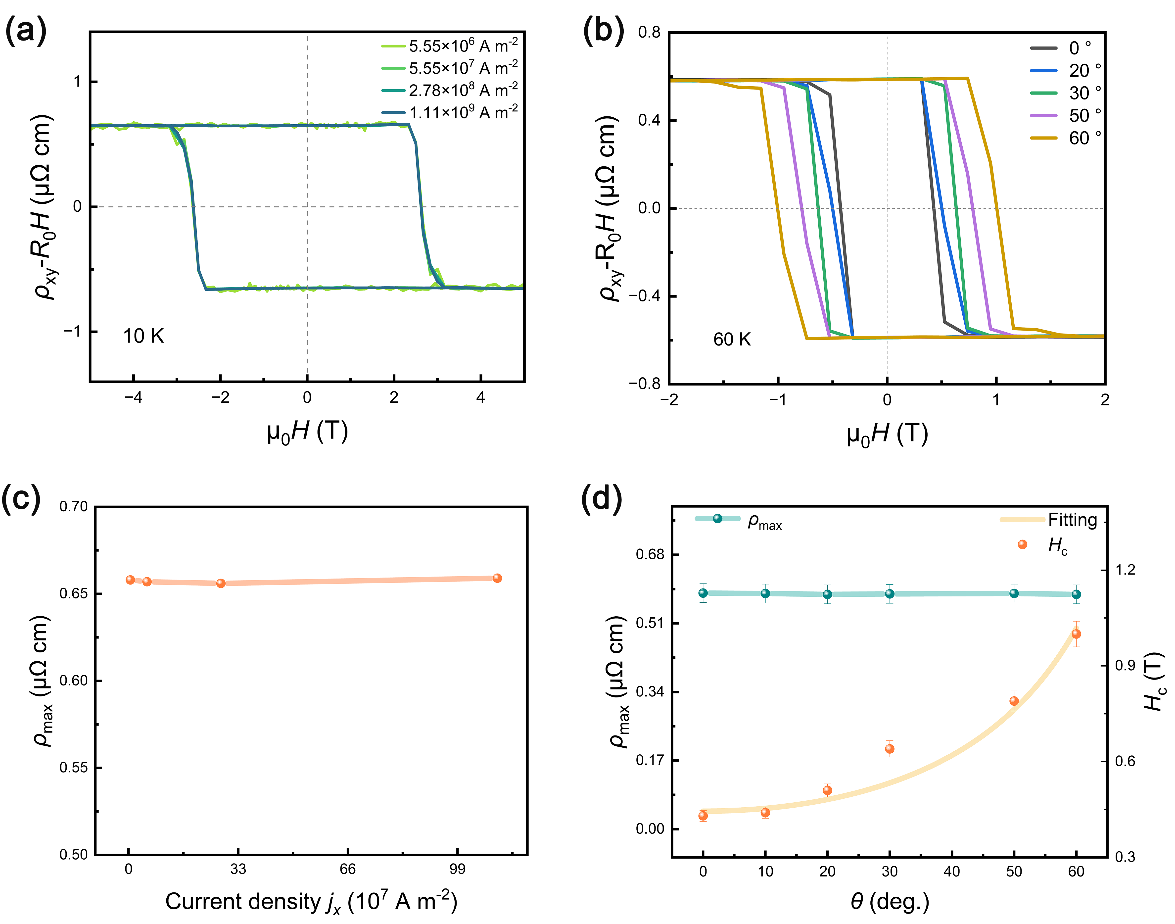
**

**Fig. S7. Robustness of AHE in RG-SRO films. (a)** Hall resistivity *ρ*_xy_ of the RG-SRO (3.6 nm) at 10 K with various current density *j*_x_. **(b)** *ρ*_xy_-*H* curves under various magnetic field canting angles *θ* at 60 K. **(c)** *ρ*_max_ as a function of current density *j*_x_ showing the robustness of the resistivity. **(d)** *ρ*_max_ and *H*_c_ as a function of canting angles *θ* demonstrating angular stability of the AHE signal. The yellow solid curve represents nonlinear fitting based on equation 1/cos(*θ*). These results highlight the fundamental distinction between conventional AHE in RG-SRO and the THE observed in freestanding membranes.


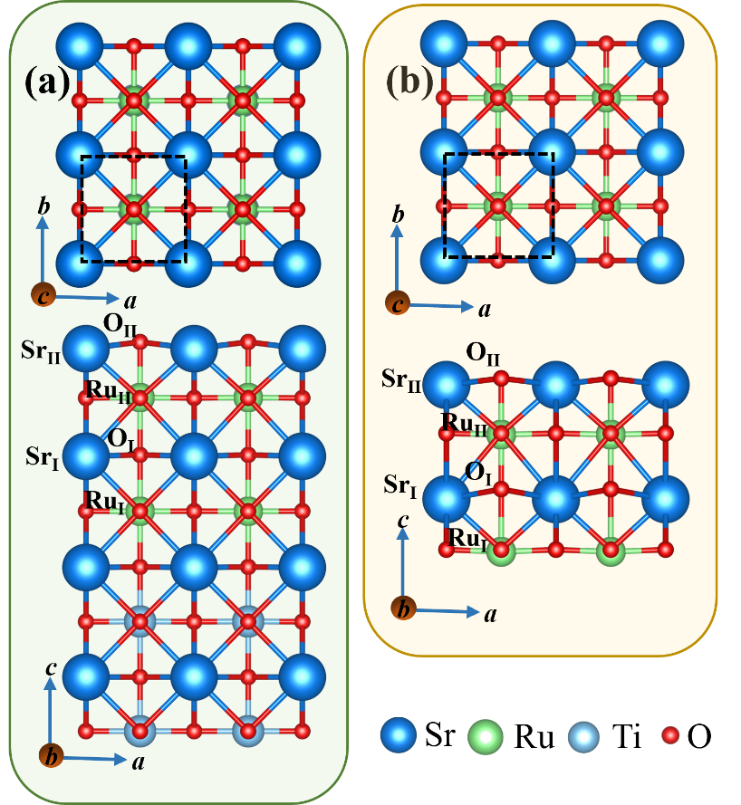


**Fig. S8.** Top and side views of structure diagrams for the RG-SRO **(a)** and FS-SRO **(b)**. The unit cell is shown by the black line.


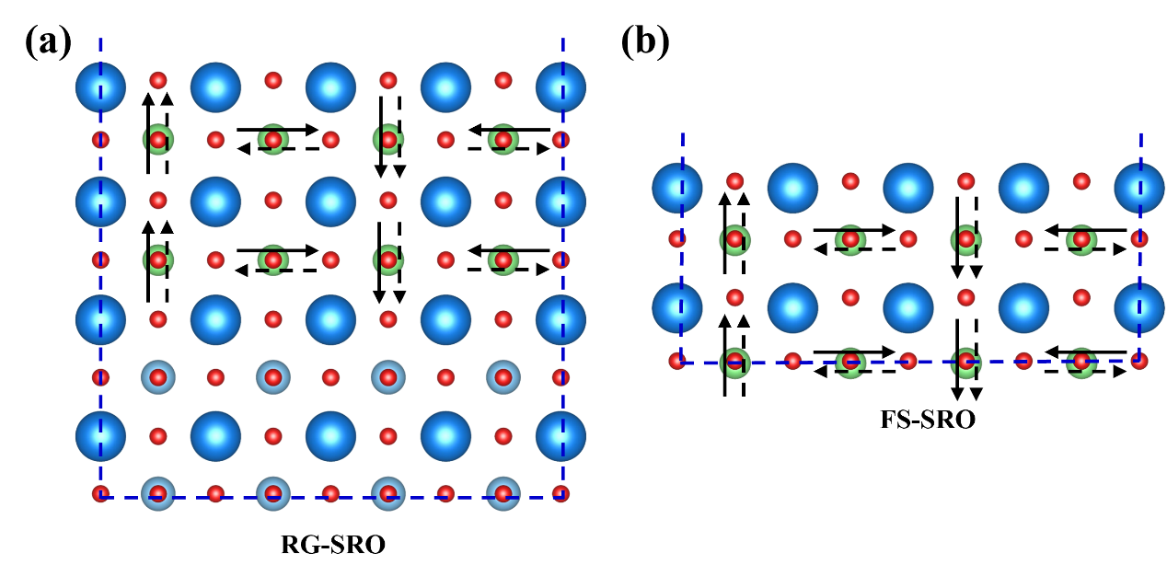


**Fig. S9.** Clockwise (solid-line arrow) and anticlockwise (dashed-line arrow) spin configurations in RG-SRO **(a)** and FS-SRO **(b)**.


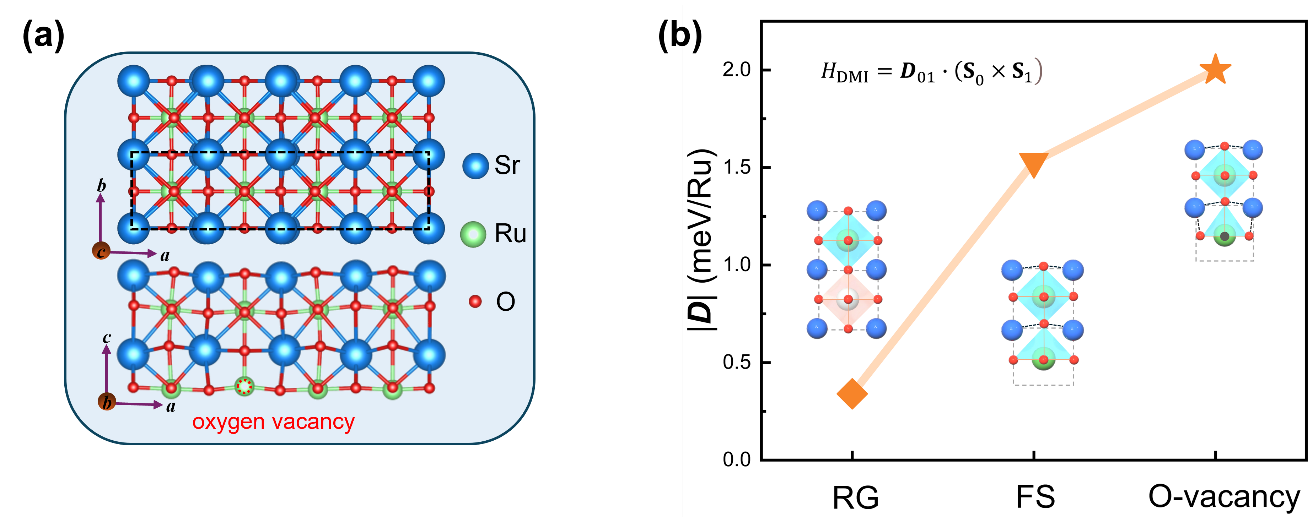


**Fig. S10.** **(a)** Top and side views of structure diagrams for the FS-SRO with oxygen vacancies. **(b)** Calculated DMI strength |***D***| for the RG-SRO, FS-SRO and FS-SRO with oxygen vacancies.


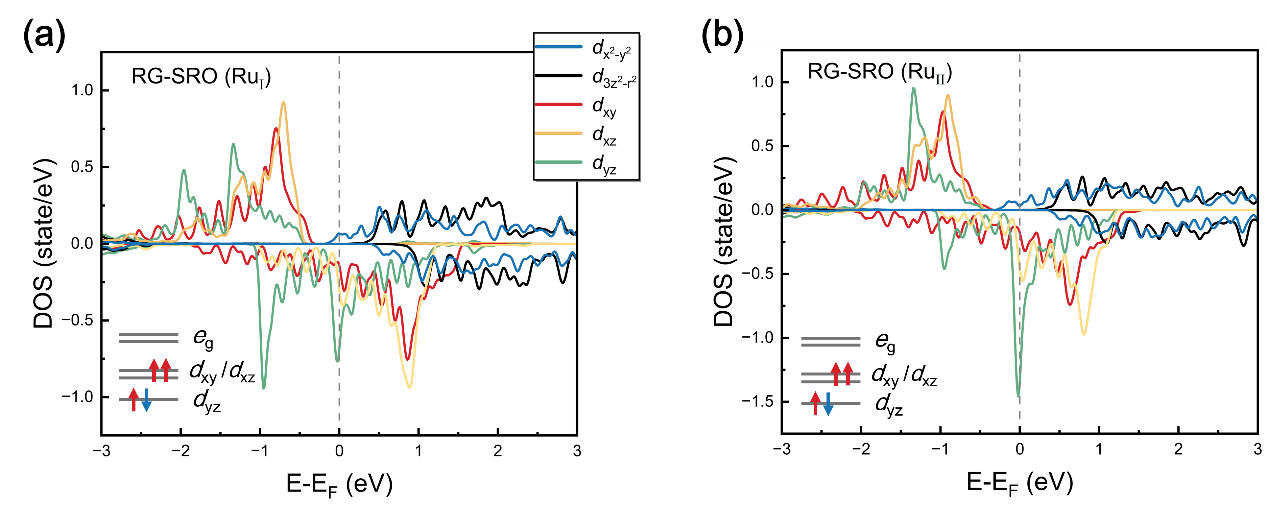


**Fig. S11.** Calculated PDOS of the Ru_I_ **(a)** and Ru_II_ **(b)** atoms in the RG-SRO. The inset is a schematic diagram of the electron configuration of the *d* orbital of the Ru atom.

**Table S1.** Calculated different Ru-O bond length (*d*_RuI-OI_, *d*_RuII-OI_, and *d*_RuII-OII_), diagonal Sr_I_-O_I_-Sr_I_ angle (∠_SrI-OI-SrI_), and localized magnetic moments of Ru_I_ and Ru_II_ atoms (*M*_RuI_ and *M*_RuII_) for the RG-SRO and FS-SRO.


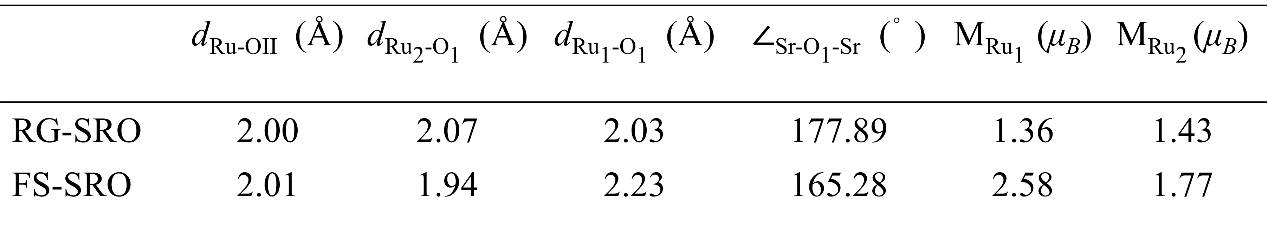


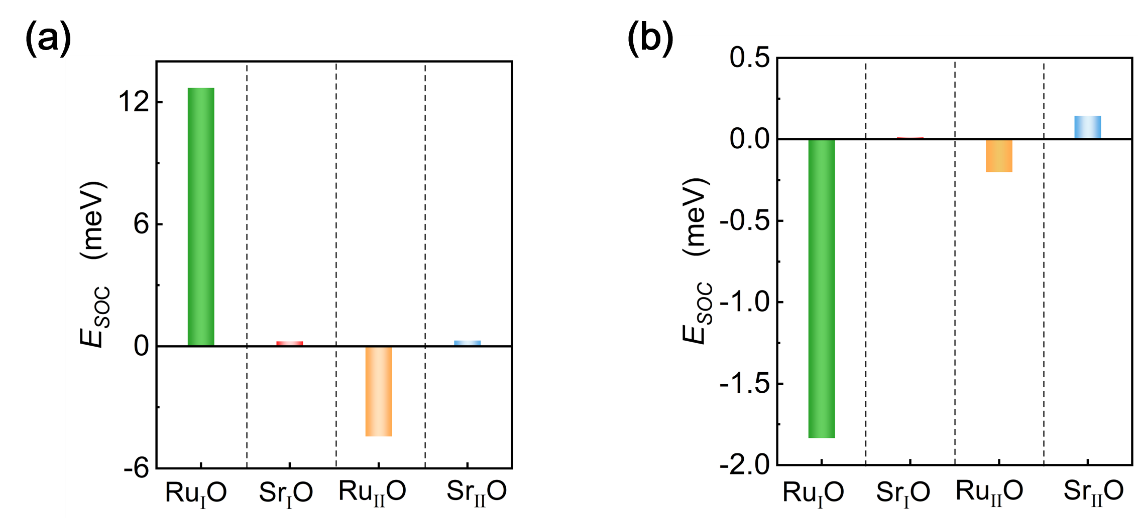


**Fig. S12.** Layer-resolved SOC energy difference (*E*_SOC_) for FS-SRO **(a)** and RG-SRO **(b)**.

**Table S2.** Conclusions of maximum topological Hall resistivity (*ρ*^T^_Max_), maximum temperature (*T*_Max_) for the existence of the THE, saturation moment (*M*_S_), DMI strength (|***D***|) and substrate in different SRO systems.

**
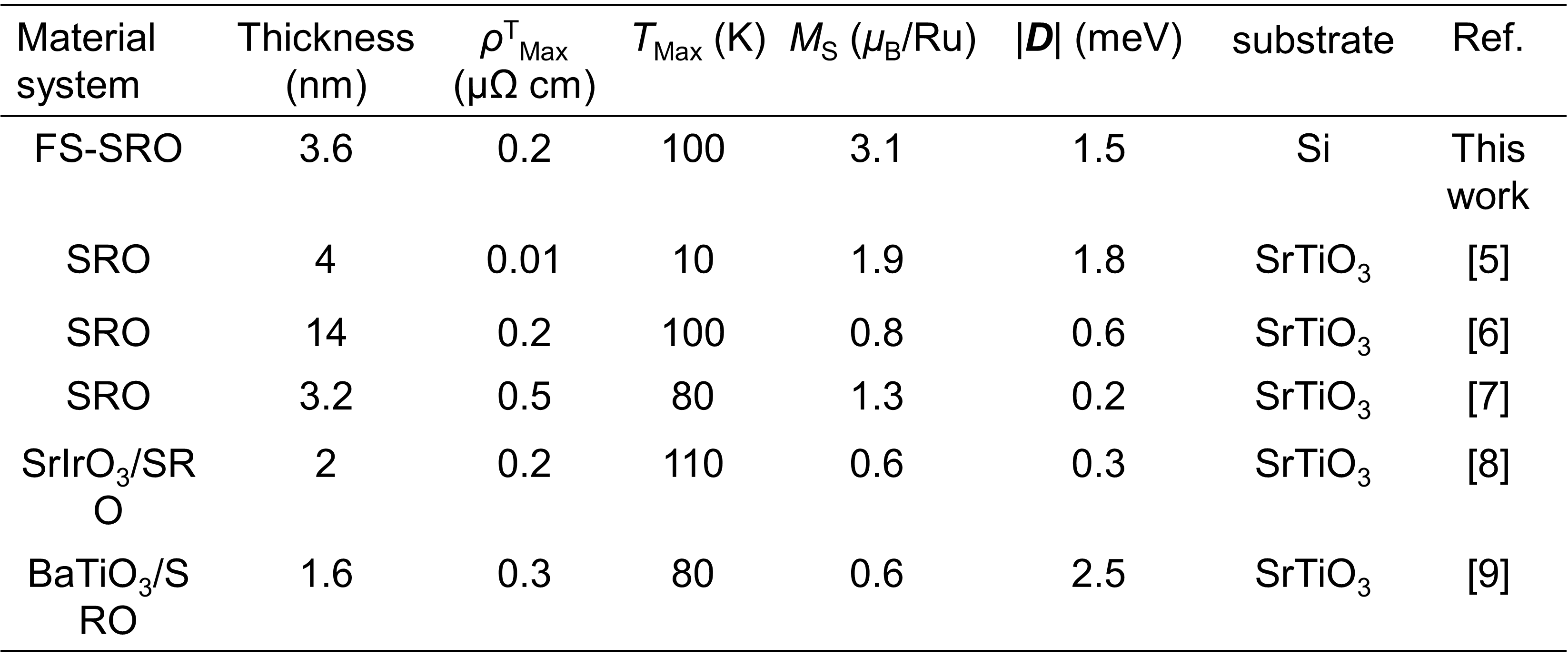
**

**References**

1. Zhang J F, Lin T, Wang A, Wang X, He Q, Ye H, Lu J, Wang Q, Liang Z, Jin F et al., Super-tetragonal Sr4Al2O7 as a sacrificial layer for high-integrity freestanding oxide membranes. Science. 2024;383(6681):388-394.

2. Lu D, Baek D J, Hong S S, Kourkoutis L F, Hikita Y, Hwang H Y, Synthesis of freestanding single-crystal perovskite films and heterostructures by etching of sacrificial water-soluble layers. Nat Mater. 2016;15(12):1255-1260.

3. Singh P, Swartz A G, Lu D, Hong S S, Lee K, Marshall A F, Nishio K, Hikita Y, Hwang H Y, Large-Area Crystalline BaSnO3 Membranes with High Electron Mobilities. ACS Appl Electron Mater. 2019;1(7):1269-1274.

4. Lu Z, Yang Y, Wen L, Feng J, Lao B, Zheng X, Li S, Zhao K, Cao B, Ren Z et al., Cooperative control of perpendicular magnetic anisotropy via crystal structure and orientation in freestanding SrRuO3 membranes. Npj Flex Electron. 2022;6(1):9.

5. Lu J D, Si L, Zhang Q, Tian C, Liu X, Song C, Dong S, Wang J, Cheng S, Qu L et al., Defect-engineered Dzyaloshinskii-Moriya interaction and electric-field-switchable topological spin texture in SrRuO3. Adv Mater. 2021;33(33):e2102525.

6. Xu Y-T, Niu X, Zhao Y-F, Zhang Y-K, Cai Y, Fu M-Y, Feng M, Qu K, Deng X, Wang B-W et al., Proton-controlled Dzyaloshinskii–Moriya interaction and topological Hall effect in hydrogenated strontium ruthenate. Mater Horiz. 2025;12:1619-1628.

7. Gu Y, Wei Y-W, Xu K, Zhang H, Wang F, Li F, Saleem M S, Chang C-Z, Sun J, Song C *et al.*, Interfacial oxygen-octahedral-tilting-driven electrically tunable topological Hall effect in ultrathin SrRuO_3_ films. *J Phys D: Appl Phys*. 2019;52(40):404001.

8. Matsuno J, Ogawa N, Yasuda K, Kagawa F, Koshibae W, Nagaosa N, Tokura Y, Kawasaki M, Interface-driven topological Hall effect in SrRuO_3_-SrIrO_3_ bilayer. *Sci Adv*. 2016;2(7):e1600304.

9. Wang L F, Feng Q, Kim Y, Kim R, Lee K H, Pollard S D, Shin Y J, Zhou H, Peng W, Lee D *et al.*, Ferroelectrically tunable magnetic skyrmions in ultrathin oxide heterostructures. *Nat Mater*. 2018;17(12):1087-1094.
